# Combinatorial delivery of CPI444 and vatalanib loaded on PEGylated graphene oxide as an effective nanoformulation to target glioblastoma multiforme: *In vitro* evaluation

**DOI:** 10.3389/fonc.2022.953098

**Published:** 2022-08-16

**Authors:** Vishnu S. Mishra, Sachin Patil, Puli Chandramouli Reddy, Bimlesh Lochab

**Affiliations:** ^1^ Department of Life Sciences, School of Natural Sciences, Shiv Nadar University, Delhi, India; ^2^ Materials Chemistry Laboratory, Department of Chemistry, School of Natural Sciences, Shiv Nadar University, Delhi, India

**Keywords:** glioblastoma multiforme, A2A receptor, drug delivery, graphene oxide, PEGylation, CPI444, vatalanib, combination therapy

## Abstract

Glioblastoma multiforme (GBM) is known as the primary malignant and most devastating form of tumor found in the central nervous system of the adult population. The active pharmaceutical component in current chemotherapy regimens is mostly hydrophobic and poorly water-soluble, which hampers clinical implications. Nanodrug formulations using nanocarriers loaded with such drugs assisted in water dispersibility, improved cellular permeability, and drug efficacy at a low dose, thus adding to the overall practical value. Here, we successfully developed a water-dispersible and biocompatible nanocargo (GO-PEG) based on covalently modified graphene oxide (GO) with a 6-armed poly(ethylene glycol) amine dendrimer for effective loading of the two hydrophobic anticancer drug molecules, CPI444 and vatalanib. These drug molecules target adenosine receptor (A2AR), vascular endothelial growth factor receptor (VEGFR), platelet-derived growth factor receptor (PDGFR), and type III stem cell receptor tyrosine kinase (c-KIT), which plays a crucial role in cancers. The effective cellular delivery of the drugs when loaded on GO-PEG is attributed to the increased permeability of the drug-nanoconjugate formulation. We observed that this combinatorial drug treatment with nanocargo resulted in a significant reduction in the overall cell survival as supported by reduced calcium levels and stem cell markers such as Oct4 and Nanog, which are two of the prime factors for GBM stem cell proliferation. Furthermore, reduced expression of CD24 upon treatment with nanoformulation impeded cellular migration. Cellular assays confirmed inhibition of cell proliferation, migration, and angiogenic potential of GBM treated with GO-PEG–Drug conjugates. Ultimately, GBM U87 cells assumed programmed cell death at a very low concentration due to nanocarrier-mediated drug delivery along with the chosen combination of drugs. Together, this study demonstrated the advantage of GO-PEG mediated combined delivery of CPI444 and vatalanib drugs with increased permeability, a three-pronged combinatorial strategy toward effective GBM treatment.

## 1 Introduction

Neuro-oncology is a challenging area dealing with malignancies of the brain and spinal cord. Among all the central nervous system (CNS) cancers, glioblastoma multiforme (GBM) is a rare-grade 4 glioma (1) that emerges from glial cells and is one of the most malignant and aggressive forms of brain tumor. The incidence rate of GBM is about 2–3 per 100,000 individuals every year, with a survival rate of around 15 months (2). The major challenge in treating GBM is their physical isolation by the blood–brain barrier (BBB), which restricts their accessibility to many drugs. Another important feature is the multilevel molecular and cellular heterogeneity (3). In particular, due to stem cell heterogeneity, GBM can overcome the loss of a cell type upon chemotherapy by other cell types and quickly evolve. Owing to the complex nature of GBM, a multifaceted approach by which divergent properties of GBM can be targeted with a better drug delivery system is the need of the hour.

Glioblastoma tumors exhibit abnormal vasculature that promotes tumor hypoxia, eventually leading to treatment resistance. One of the major players, vascular endothelial growth factor (VEGF), contributes to the abnormal vasculature and could be a prime target to treat GBM (4). VEGF and its signaling pathway have been most extensively explored in context with angiogenesis in tumors, including GBM (5), and have been targeted by various chemotherapeutic drugs like tivozanib, sorafenib, and others. Bevacizumab, a humanized mAb (mono clonal antibody) against VEGF, is the first and only FDA (U.S. Food and Drug Administration) approved immunotherapy for GBM. However, it has been suggested that bevacizumab increases progression-free survival but does not significantly increase the overall survival of the patients (6). Another anti-angiogenic drug, vatalanib (PTK787), is a tyrosine kinase inhibitor of all VEGF (VEGFR-1, -2, and -3) receptors, platelet-derived growth factor receptor-*β* (PDGFR-*β*), and proto-oncogene, c-kit (4, 7). This drug has been shown to be well tolerated by cancer patients (8, 9). A recent study using vatalanib in a mouse model for Alzheimer’s disease (AD) has demonstrated its capability to cross the BBB and cause reduced AD pathology (10). A previous report also indicated that vatalanib can be effective in treating gliomas (11). These studies suggest that vatalanib could be effective in targeting the characteristic angiogenic properties of GBM along with a suitable partner to tackle heterogeneous nature of GBM.

Another emerging target of various cancers is the A2A adenosine receptor (A2AR). It is a guanine nucleotide-binding protein, and a G-protein coupled adenosine receptor and acts through mitogen-activated protein kinase (MAPK) and protein kinase A (PKA), signaling converging at the cyclic adenosine monophosphate (cAMP) response element (CREB) (12). This signaling axis regulates various cellular processes such as neuronal plasticity, neuroprotection, vasodilation, and immunosuppression (13). In glial cells, purinergic signaling has been shown to be modulated by various stress factors such as hypoxia, pro-inflammatory cytokines such as tumor necrosis factor-α (TNF-α), interleukin-1β (IL-1β) by elevated A2A receptor (13). These dynamics in A2A receptor mediated signaling affect various cellular processes of glial cells such as proliferation, differentiation, and apoptosis (14). Among these, targeting the immunosuppressive properties of purinergic signaling has been explored as a potential cancer therapy by using various antagonists to this pathway (15–17). Moreover, due to the neuronal origin of GBM, targeting A2A receptor mediated signaling may also affect different cellular functions. Therefore, choosing the A2A receptor antagonist might prove a double-edged sword.

CPI444, an antagonist of the human A2A receptor (18), is one of the earliest drugs evaluated in cancer therapies (19, 20). This drug has shown promising antitumor activity by increasing the CD8+ (cluster of differentiation 8 positive) T-cell infiltration due to blockage of adenosine activity (20). CPI444 also exhibited 66-fold higher inhibition specificity in A2A receptor over the A1 receptor (18). A recent report indicates the promising tolerance of this drug by renal cancer patients (19). Moreover, a recent study by Corvus Pharmaceuticals, Inc. has shown a minimum of 15% of CPI444 crossing BBB (21). Therefore, CPI444 is a viable choice to targeting divergent cellular mechanisms associated with GBM.

Nanoparticles have been widely explored as a carrier in the past few decades to improve the efficacy and reduce the side effects of anticancer drugs. Graphene and functionalized graphene derivatives offer unique physico-chemical properties and are the most widely explored nanocargos to deliver payloads such as drugs, nucleotides, proteins, etc. (22, 23). Graphene assists in excellent loading of hydrophobic cancer drugs by either non-covalent or covalent interactions due to its two-dimensional planar nanoarchitecture and a very high specific area. The ease of its preparation *via* chemical oxidation of graphite, followed by exfoliation to form graphene oxide (GO) nanoflakes, resulted in well-exposed oxygen-enriched functionalities such as hydroxyl, carbonyl, carboxyl, and epoxide. These multiple surface functionalities on GO provide excellent aqueous dispersion stability and scope of derivatization to impart biocompatibility and tuning capabilities to modulate charge, size, etc., essential for the delivery of anticancer drugs. In the past, several efforts have been made to amplify biocompatibility, aqueous stability and opsonization with a prolonged blood circulation time of GO, by functionalization with poly(ethylene glycol) (PEG), FDA approved polymer (24, 25). There are several reports on using GO-PEG to deliver several cancer drug molecules such as doxorubicin, cis-platin, camptothecin, etc. However, single drug-based conventional therapeutic approaches are not successful due to the complexity of the tumors and may cause drug resistance. Usage of multiple drugs is an attractive option where each component has a specific role to play and mitigates heterogeneity associated with the tumors. Various physical properties of drugs, such as molecular size, solubility, stability, and cellular delivery, are an important aspect of combinatorial drug treatment. However, their optimal dosage reaching the tumor site is essentially important for effective cancer treatment. This demands exploration of rational design of nanocarriers which can efficiently load multiple drugs and assist cellular delivery in the desired ratio to enable synergistic interactions, if any, with improved chemotherapeutic potential. Additionally, independent studies have demonstrated that GO-based nanostructures, including GO-PEG, can cross the BBB (26, 27) and are used in drug delivery for tageting glioma (28). This property proves invaluable, especially in the treatment of GBMs where crossing the BBB is a major hurdle for many chemotherapeutic agents.

In this work, a GO-PEG nanodrug delivery system is designed, synthesized, and characterized for the combinatorial therapy of two drug molecules, CPI444 and vatalanib. The drug molecules are separately loaded on GO-PEG and then combined in an optimal ratio to form a nanoformulation. The effective ratio of the two drugs was predetermined using a carrier-free *in vitro* experiment in the GBM U87 cell line. The current combinatorial therapeutic approach may provide new avenues and directions to the forefront of several therapeutic and developmental chemotherapy regimens.

## 2 Materials and methods

### 2.1 Materials

Graphite flakes (Alfa Aesar, 99.8%, natural graphite 325 mesh), 6-arm PEG-amine (JenKem Technology, 15 kDa), sulfuric acid (H_2_SO_4_, Rankem, 95%−98%), potassium permanganate (KMnO_4_, Aldrich, 99%), phosphoric acid (H_3_PO_4_), hydrogen peroxide (30% w/v) and potassium hydroxide (KOH, powder) from Finar Scientific, Vatalanib (cat#A3969, APExBIO), CPI444 (cat#1202402-40-1, MedChemExpress), and GBM U87 cell lines were obtained from the National Centre for Cell Sciences (NCCS) Pune. The origin of GBM U87 (or U-87 MG) is a malignant glioma from a human male patient with epithelial morphology. It is from the primary brain tumor and grade IV glioma according to the WHO classification (29). MEM (Minimum Essential Medium Eagle, Cat#AL080A) media, trypsin-EDTA solution 1× 0.25% (Cat#TCL007), FBS (Fetal Bovine Serum, Cat#RM10681), antibiotic solution (penicillin and streptomycin), and PBS (Dulbecco’s Phosphate Buffered Saline, powder pH 7.4) were purchased from HiMEDIA, India. Fluorescein isothiocyanate (FITC, Invitrogen) and CCK-8 (Cell Counting Kit-8/Cytotoxicity assay kit) were purchased from Dojindo Molecular Technologies, USA.

### 2.2 Characterization

A Thermo Scientific Evolution 201 UV−visible spectrophotometer was used to measure the absorbance in a 1 cm quartz cuvette over the range of 200−800 nm. A Nicolet iS20 mid-infrared FTIR spectrometer equipped with an interferometer with KBr/Ge coated beam splitter, deuterated triglycine sulfide (DTGS) detector, and attenuated total reflectance diamond (iD5-ATR) accessory was used to measure the Fourier transform infrared (FTIR) in the range of 4,000 to 450 cm^−1^ with a resolution of 0.25 cm^−1^. A Jobin Yvon HR800 Raman microscope was used for Raman analysis of nanomaterials at a wavelength (λ) of 514 nm. Disorder and defects in graphitic materials were measured by the ratio of the intensity of D and G bands, i.e., (*I*_D_/*I*_G_). Structure disorder in the graphite network raises the intensity ratio and is inversely proportional to the average sp^2^ cluster size. The distance between defect points (*L*_D_) in the GO samples was calculated from the intensities of the D (sp^2^-hybridized carbon breathing mode) and G (graphitic sp^2^-hybridized carbon) bands in the Raman spectra using the Tuinstra–Koenig relation, (C × λ = 102 nm^2^), using Equation (1) (30).


(Equation 1) $$L_{D}=\sqrt{\left(C\times \lambda \right)\left(I_{G}/I_{D}\right)}$$

The average crystallite size (*L*_a_) of the sp^2^ domains in the synthesized GOs was determined by Equation (2),


(Equation 2) $$L_{a}=\left(2.4\times 10^{-10}\right)\lambda^{4}{\left(\frac{I_{D}}{I_{G}}\right)}^{-1}$$

A Rigaku Smart Lab X-ray diffractometer was used to study the powder X-ray diffraction (PXRD) of nanoparticles using Cu-K_α_ radiation (λ = 0.154 nm) over a 2θ scan range of 4° to 60°, where θ is the Bragg angle. A Mettler Toledo thermogravimetric analyzer (TGA) with a built-in gas controller (TGA2 SF/1100) and fitted with an XP1U TGA balance (ultra-micro balance) was used to study the thermodynamic properties of nanoparticles in a temperature range from 50 to 700°C in a nitrogen atmosphere at a flow rate of 50 ml min^−1^ and a heating rate of 10°C min^−1^. The surface chemistry of nanomaterials was analyzed by X-ray photoelectron spectroscopy (XPS) (Omicron multiprobe surface analysis system) using a monochromatized AlK_α_ (1,486.7 eV) radiation source. A dynamic light scattering (DLS) instrument (Nanosizer, Malvern, UK) was used to measure hydrodynamic particle size and zeta potential (ζ) using an argon laser at a λ = 633 nm, with a detector angle of 90°, at room temperature.

### 2.3 Preparation of graphene oxide (GO)

GO was prepared from graphite powder using a previously reported method (23). Graphite powder (1.0 g) was oxidized by a mixture of concentrated sulfuric acid and phosphoric acid (134:15 ml, v/v) followed by the addition of potassium permanganate (6.0 g) at room temperature. The reaction mixture was stirred at 50°C for 12 h, and after that allowed to cool to room temperature before the slow addition of a mixture of ice-cold water: H_2_O_2_ (250:3 ml) with vigorous stirring. A color change from dark brown to yellow is noticed. Upon gravity settling, GO particles settled down and the supernatant was decanted. The so-obtained residue was washed multiple times with water and GO as the residue was separated by centrifugation (Eppendorf 5425 R) at 11,000 rpm. This process was repeated until the pH of the supernatant became neutral. Finally, the brown slurry obtained was dried under vacuum on a rotary evaporator at 50°C and GO was obtained as a black powder (2.3 g).

### 2.4 Preparation of PEGgylated graphene oxide (GO-PEG)

An aqueous dispersion of GO (0.5 mg/ml, 20 ml) was prepared in deionized water by probe sonication for 2 min (Sonics USA, 500 W, 20 kHz) at 40% maximum amplitude and a pulse rate of 10 s (on and off) followed by bath sonication for 2 h. To this GO dispersion, 6-armed PEG amine (60 mg) and KOH (80 mg) were added. The reaction mixture was stirred vigorously at 80°C for 24 h. Finally, GO-PEG was obtained by purifying the reaction mixture by dialysis membrane (mol. weight cut-off = 10 kDa) against deionized water for 24 h to remove the excess or unbound 6-armed PEG amine. The GO-PEG dispersion was stored at 4°C.

### 2.5 Preparation of CPI444@GO-PEG and vatalanib@GO-PEG

A stock solution of CPI444 (1.1 mg) was prepared in a cosolvent mixture of water and tetrahydrofuran (1:1 v/v, 2 ml) and mixed with the prepared dispersion of GO-PEG (0.5 mg/ml, 2 ml). The resultant mixture was stirred vigorously at room temperature for 24 h. Excess or unbound CPI444 was removed by dialysis membrane (mol. weight cut-off = 10 kDa) against deionized water (50 ml × 4) for 24 h. Removal of excessive unbound amounts of CPI444 on GO-PEG was monitored and calculated using UV–visible spectroscopy at a maximum absorption wavelength (*λ*_max_) of CPI444 at 356 nm. The so-formed CPI444@GO-PEG nanoconjugate was stored at 4°C for further study. Similarly, vatalanib@GO-PEG was also prepared following the same method. In brief, a stock solution of vatalanib (1.01 mg) was prepared in a cosolvent mixture of water and tetrahydrofuran (1:1 v/v, 2 ml) and mixed with the prepared dispersion of GO-PEG (0.5 mg/ml, 2 ml) and dialyzed. For CPI444 + vatalanib@GO-PEG, optimal amounts of CPI444@GO-PEG and vatalanib@GO-PEG (with the same drug combination, 1:1 molar ratio, as without GO-PEG) were premixed and probe sonicated for 2 min to homogenize, just prior to study.

### 2.6 Drug loading capacity on GO-PEG nanocarrier

To determine the loading of CPI444 on GO-PEG, UV–vis spectroscopy measurements were carried out. A standard curve of CPI444 (linear fitting with *R* ^2^ = 0.99505) at different concentrations was plotted at a *λ*_max_ of 356 nm. After the reaction completion of the CPI444@GO-PEG preparation, an unbound or excess amount of leached out CPI44 drug, during dialysis, was calculated using a standard curve and subtracted from the added amount of drug. The percentage loading and encapsulation efficacy were calculated using Equation (3) and Equation (4), respectively.


(Equation 3) $$Drug\;loading\;\left(\%\right)=\;\frac{Drug\;adsorbed\;on\;GO-PEG\;\left(\mu g\right)}{GO-PEG\;added\;\left(\mu g\right)}\times 100$$

(Equation 4) $$Drug\;encapsulation\;efficacy\;\left(\%\right)\;=\;\frac{Drug\;adsorbed\;on\;GO-PEG\;\left(\mu g\right)}{Drug\;adeed\;\left(\mu g\right)}\;\times 100$$

Similarly, the percentage loading and encapsulation efficacy of vatalanib on GO-PEG were determined using the same protocol as in the case of CPI444 on GO-PEG, by UV–vis spectroscopy. The standard curve of vatalanib (linear fitting with *R* ^2^ = 0.99665) at different concentrations was plotted at a *λ*_max_ of 335 nm.

### 2.7 Cell culture conditions

Glioblastoma multiforme (Human) cancer cell line GBM U87 was cultured in Minimum Essential Media (MEM) containing 10% FBS and 1% antibiotic (penicillin/streptomycin) solution in a humidified cell culture incubator (Galaxy 170R, Eppendorf) containing 5% CO_2_ at 37°C.

### 2.8 Cell viability study

The cell viability study of pristine drugs (CPI444, vatalanib, and CPI444 + vatalanib), GO-PEG, and respective drug–GO-PEG nanoconjugates (CPI444@GO-PEG, vatalanib@GO-PEG, and CPI444 + vatalanib@GO-PEG) were assessed using the CCK-8 kit (Cat#CK04, Dojindo). The GBM U87 cells were seeded into a fresh 96-well flat-bottom plate at a density of 5,000 cells/well. After cells were attached for 18–24 h, they were treated separately with pristine drugs (2–60 µM), GO-PEG (2–20 µg/ml), and nanoconjugates (with a drug loading of 1–30 µM) at different concentrations for 48 h. After the treatment, 10 µl of CCK-8 solution was added to each well. Optical density (OD) at a *λ* of 450 nm was recorded on a microplate reader (BioRad). All the experiments were repeated three times independently. The relative percentage of absorbance was calculated, considering control as cent percent.

### 2.9 Cellular uptake of GO-PEG

GBM U87 cells were seeded in a 60 mm culture dish and treated with FITC-tagged GO-PEG with a concentration of 0 µg/ml (untreated negative control), 5 µg/ml, 10 µg/ml, and 15 µg/ml. After 24 h, cells were harvested using trypsin. Cells were washed with 1× PBS twice and then resuspended in 500 µl of 1× PBS + 0.1% FBS solution. The cells were analyzed using a CytoFLEX flow cytometer and 10,000 events were acquired for each sample.

### 2.10 Fluorescent activated cell sorting (FACS) analysis

GBM U87 cells were seeded in a 60 mm dish and, after 18 h, cells were subjected to target drug formulations with appropriate controls. After 48 h of treatment, the cells were harvested and processed for FACS analysis.

#### 2.10.1 Cellular calcium level quantitation by Fuo-4AM fluorophore

Harvested cells were incubated in 3 µM concentration of Fluo-4AM dye (cat#F14201, Life technologies) in dark at room temperature for 20 min. After incubation, cells were washed twice with 1× PBS and resuspended in 500 µl of 1× PBS solution with 0.1% FBS solution.

#### 2.10.2 CD24 marker staining

Here, cells were washed twice with 1× PBS and then resuspended in a 1× PBS solution with 0.1% FBS. Cells were stained with CD24 mAb-FITC (cat#MHCD2401) at a concentration of 1:100 for 45 min in the dark at room temperature. Cells were collected and resuspended in 500 µl of 1× PBS solution with 0.1% FBS solution.

The cell suspensions were analyzed using a CytoFLEX flow cytometer and 10,000 events were acquired for each sample.

### 2.11 Transwell-chamber migration assay

Transwell inserts (24 well, 8 μm pore size; HiMEDIA) were used to assess the migratory abilities of cells. Pre-treated GBM U87 cells with GO-PEG, CPI444 + vatalanib, and CPI444 + vatalanib@GO-PEG along with untreated control for 48 h were used for this assay. The treated cells were suspended at a final density of 3.5 × 10^4^ cell/ml in a serum-free medium and seeded in the upper-well of the chamber. The lower-well of the chamber contained media supplemented with 10% FBS. After 24 h, cells on the surface of the upper-well were removed by a cotton swab. Cells that had migrated through the filter to the lower chamber were fixed in 4% paraformaldehyde and stained with 1% crystal violet. Cells were counted from three randomly selected fields per each chamber under a microscope (ECLIPSE Ti, Nikon).

### 2.12 Cell adhesion assay

Cells were seeded in a 60 mm dish and treated with drugs as mentioned above for 48 h. Cells were harvested and re-suspended in media. Meanwhile 12-well plate coated with 50 μM poly-L-lysine (cat#P3513, Sigma) for 1 h at room temperature. Treated/untreated GBM U87 cells were harvested and suspended at a final density of 3 × 10^5^ cells/ml and plated on poly-L-lysine-coated plates for 30 min. After that, unattached cells were removed by inverting the 12-well plate and gently washing twice with 1× PBS. The attached cells were fixed in ice-cold methanol for 10 min at room temperature. After a wash with 1× PBS, cells were stained with 1% crystal violet stain for 5 min with gentle shaking. The plate was washed with water and kept for drying. After that, images were captured by a color camera. Later, stain was solubilized into 1 ml of 1% sodium dodecyl sulfate (SDS) for 1 h and collected in fresh 1.5 ml centrifuge tubes. Absorbance was recorded at 595 nm (Bio-Rad microplate reader) in a 96-well plate in four replicates. The absorbance value of the control sample was converted to 100% and relative percentage changes in other treatment conditions were calculated.

### 2.13 Invadopodia assay

GBM U87 cells were harvested and seeded in a 60 mm dish for the invadopodia experiment. The cells were treated with specific drugs and GO-PEG–drug conjugates for 48 h following the procedure mentioned above. Prepared fluorescent gelatin-coated coverslips according to the protocol (31). Briefly, GBM U87 cells were harvested and plated on coverslip in 60%–70% confluency which was already coated with gelatin labeled with oregon green 488 (cat#G13186, Life technologies). After that, the coverslip with the cells was transferred to a CO_2_ incubator for 15 h for the initiation of the gelatin degradation. After 15 h of incubation, the coverslip was taken out of the plate and quickly fixed with 4% formaldehyde for 10–15 min at room temperature. The fixation solution was removed and washed with 1× PBS twice. Blocking and permeabilization were performed with a solution (3% BSA in 1× PBS containing 0.1% Triton X-100) for 15–20 min at room temperature in the dark. The blocking solution was removed and washed with 1× PBS twice. This was probed with Alexa Fluor 555 Actin-Phalloidin (1:500 dilution) for 30–40 min in the dark. Actin-Phalloidin was removed and washed with 1× PBS twice, and after that, the coverslip was mounted by inverting it over a glass slide containing a drop of mounting medium ProLong™ Gold antifade reagent with Hoechst (Invitrogen). The slides were dried and sealed with colorless nail paint. Images were taken under a Nikon Fluorescent Microscope (at ×60) for visual comparison.

### 2.14 Colony forming assay

To perform a 2D colony forming assay, cells were seeded in 6-well plates and then treated with various combinations of test drugs for 48 h. After 48 h, treated/untreated cells were placed in fresh 6-well plates at a cell density of 1,000 cells/well. Every third day, the media was replenished until control well-achieved confluency. The experiment was observed for up to 12 days and images were captured under a microscope (DIC imaging, ECLIPSE Ti, Nikon). Then, cells were fixed in 4% paraformaldehyde for 10 min and stained with crystal violet stain for 10 min. After washing the plates with water slowly, cells were collected in 1% SDS and absorbance was measured at 595 nm. The relative absorbance was compared to control sample in percentages (control was treated as cent percent).

### 2.15 Tube formation assay and proteome array

The tube formation assay and cytokine array analysis were conducted as described previously (32). Briefly, primary endothelial cells were cultured and incubated with conditioned media of treated and untreated cells. Tube formation was analyzed after 4 h and images were captured. The number of branches and nodes was calculated manually and the same was shown as bar plots. For proteome array analysis, total cell lysate of all treated cells was collected and quantified by Bradford reagent. An equal amount of protein was loaded on each membrane for 24 h and developed as per the protocol of the manufacturer (cat#ARY010, R&D systems).

### 2.16 Western blot analysis

The effect of treatment of CPI444 and vatalanib@GO-PEG on apoptotic markers (proteins) was tested. Here, after 48 h of treatment with test drug formulations and appropriate controls, total cell lysate was prepared and quantified using Bradford reagent. Proteins were separated by 10% sodium dodecyl sulfate–polyacrylamide gel electrophoresis (SDS-PAGE) for 3 h. The proteins were transferred onto the polyvinylidene fluoride (PVDF) membrane after blocking blots were incubated with primary antibodies overnight at 4°C. The blots were washed and developed using appropriate horseradish peroxidase (HRP)-conjugated secondary antibodies following a standard protocol using chemiluminescent substrate (SuperSignal™ West Pico PLUS, cat#34580, ThermoFischer). The images were acquired by the FluorChem E system and densitometry was carried out by ImageJ software.

Primary antibodies: anti-PARP in Rabbit, cat#ITM3132; Anti-Bcl2 in Mouse, cat#ITT5756 and anti-β-actin in Rabbit from G-biosciences

Secondary antibodies: HRP-Goat-anti-Rabbit, cat#SE134 from G-biosciences and HRP-Goat-anti-Mouse, 554002 from BD biosciences.

### 2.17 Statistical analyses

The statistical analyses were performed using the GraphPad Prism Version 7.04 software. Here, the Student’s two-tailed *t*-test was performed using the mean from the minimum of three independent observed values, and respective *p*-values are reported.

## 3 Result and discussion

### 3.1 Synthesis and characterization of nanoparticles

**Figure 1:**
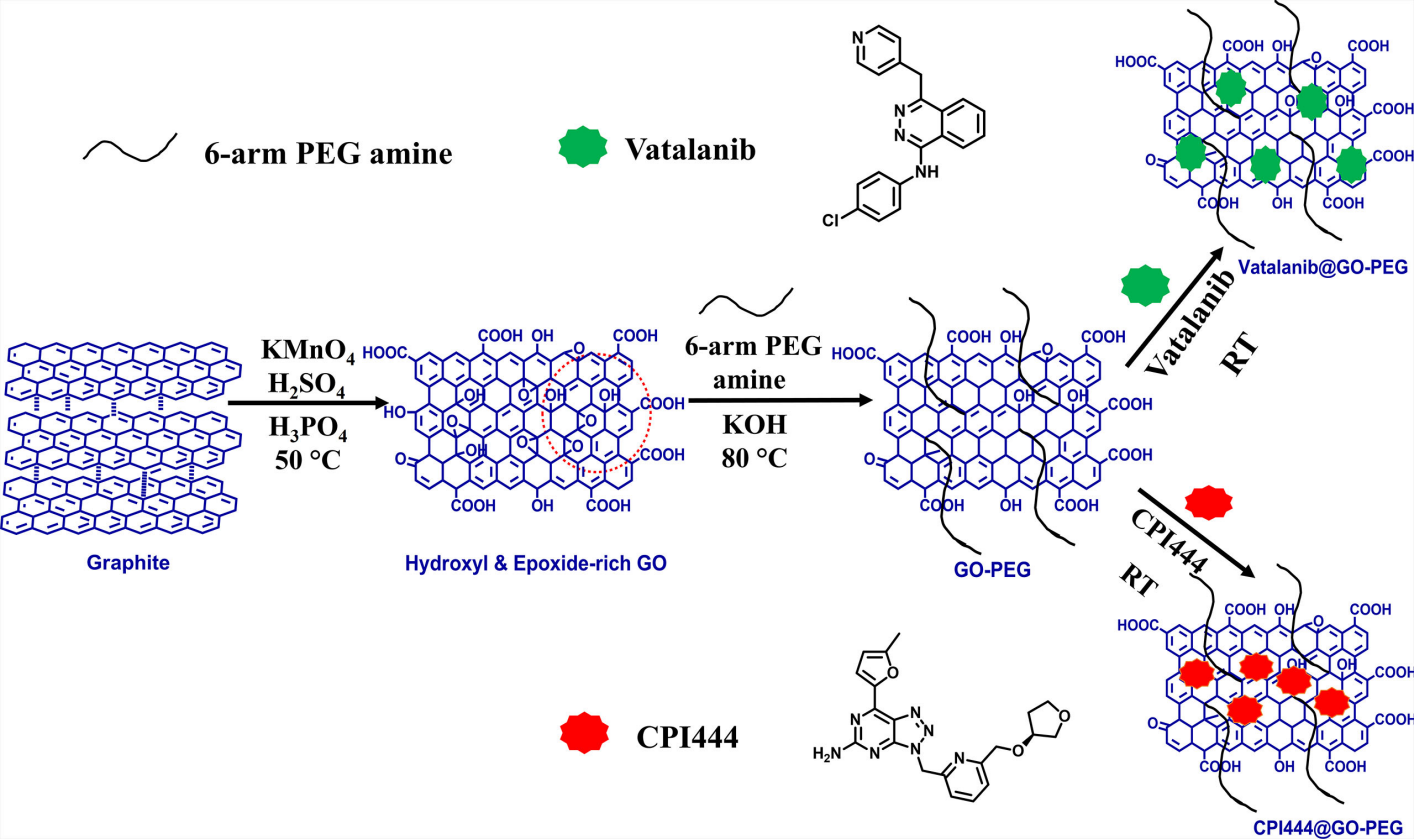
Schematic diagram for the synthesis of GO, GO-PEG, and drug loaded GO-PEG nanocarriers (CPI444@GO-PEG and vatalanib@GO-PEG).

**Figure 2:**
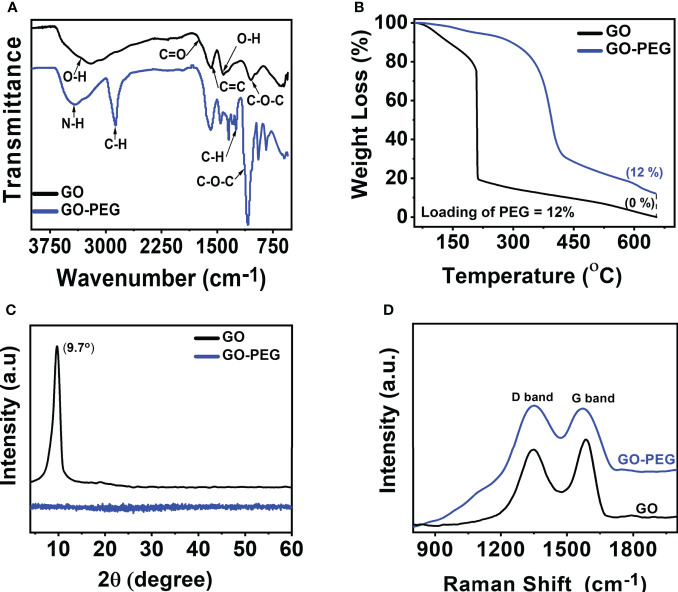
Characterization of GO and GO-PEG nanoparticles. **(A)** FTIR, **(B)** TGA, **(C)** PXRD, and **(D)** Raman spectra.

The preparation of GO-PEG from graphite proceeds in two steps, as illustrated in **Figure 1**. In the first step, graphene oxide (GO) was prepared from graphite using the modified Hummer’s method. This involved chemical oxidation of graphite by acidified potassium permanganate. This method provides the benefit of keeping the graphitic planes relatively intact, and oxidation occurs mainly on the edges to introduce oxo-functionalities. The fewer defect in the basal plane ensures swift binding and efficient loading of the highly aromatic drug molecules *via* π-π stacking. The oxygen-rich functionalities such as carboxyl and hydroxyl groups are necessary to improve the aqueous dispersion stability of the GO nanoparticles, while epoxide and carbonyl groups on the basal plane are desired for the covalent modification of the GO (24). In the second step, the epoxide and carbonyl groups on GO were reacted with the 6-armed PEG-amine to form PEGylated graphene oxide, GO-PEG. The reaction is favored in alkaline medium and proceeded by base mediated nucleophilic ring-opening and addition reaction. This led to the enrichment of the surface of nanoparticles with an enormous number of covalently-linked free amine groups, in addition to the existing carboxyl and hydroxyl functionalities. As a result, the subsequent increase in polar functionalities in GO-PEG occurs to offer hydrophilic properties to the prepared nanocarrier. Following the purification, both GO and GO-PEG were characterized by various techniques. **Figure 2A** shows the FTIR spectra of GO and GO-PEG. The successful synthesis of GO from graphite was confirmed by the presence of oxygen-rich functionalities. The characteristic peaks at 3,400 cm^−1^, 1,740 cm^−1^, 1,590 cm^−1^, 1,412 cm^−1^ and 1,050 cm^−1^ are assigned to the O–H (of carboxylic group), >C=O (carbonyl), C=C (aromatic, sp^2^ hybridized carbon), O–H (adsorbed water) and C–O–C (epoxide ring) vibrations, respectively. The FTIR spectrum of GO-PEG is grossly dissimilar to GO, and clearly the carbonyl-related stretch is relatively vanished. Additionally, several new IR vibrations at 3,390 cm^−1^, 2,868 cm^−1^, and 1,250 cm^−1^ for the N–H stretching, C–H stretching, and C–H deformation vibrations, respectively, are observed. It is evident that the intensity of C–O–C stretching vibration at 1,050 cm^−1^ substantially increased in GO-PEG, which is due to the large number of ether linkages in the PEG structure. This demonstrated the presence of PEG-amine bound to GO in the synthesized nanocarrier.

To determine the amount of PEG-amine coating on GO, thermogravimetric analysis (TGA) was performed. From **Figure 2B**, GO showed mass loss in the range of 80 to 220°C associated with the evaporation of adsorbed water and thermodynamic decomposition of highly labile oxygen functionalities (33). GO revealed a residual char yield of 0% at 650°C. Unlike GO, GO-PEG is relatively more thermodynamically stable and showed an initial mass at a high temperature of ~315°C, which clearly demonstrated the existence of a polymer coating on GO sheets (34). Comparison of residual char yield obtained at 650°C in GO and GO-PEG revealed a ~12% loading of PEG-amine on GO.

The powder X-ray diffractogram (PXRD) spectrum (**Figure 2C**) of GO showed the absence of graphite (002) peak at 2θ = 26.28° (35), but a new (001) peak at low 2θ = 9.7° clearly supports the successful oxidation of graphite to GO and the absence of graphite impurity in it. Surprisingly, no diffraction peaks are observed in GO-PEG, including the disappearance of diffraction peaks related to GO, which suggests PEG coating resulted in the formation of amorphous GO-PEG nanoparticles. The interlayer spacing of the materials is proportional to the degree of functionalization and inversely related to the observed 2θ. GO showed an interlayer spacing of 9.1 Å (>3.4 Å in graphite), calculated using Bragg’s equation (36) suggesting significant oxidation occurred in graphite to form GO. GO-PEG diffractogram remained featureless, suggesting the interlayer spacing may be further enhanced due to the introduction of extra PEG-amine coating on the GO-PEG nanostructure.

The Raman spectra as shown in **Figure 2D** show D and G bands centered at ∼1,350 cm^−1^ and ∼1,590 cm^−1^, respectively, confirming the typical lattice distortions (37) in GO. Defects in graphitic materials can be assessed by evaluating the ratio of intensities in the D and G bands, i.e., the *I*_D_/*I*_G_ ratio. A higher *I*_D_/*I*_G_ (1.04) in GO-PEG than that observed in GO (0.907), along with a subsequent broadening of bands, signifies an increased defect ratio of GO upon functionalization with the dendrimer. Interestingly, since PEGylation occurred on the pre-existing epoxide and carbonyl defects in GO, both the nanoparticles demonstrated an identical distance between defects (*L*_D,_ ~10 nm). GO-PEG revealed a reduced in-plane crystallite size (*L*_a_) from 18.5 nm to 16.1 nm, supporting PEGylation induced shrinking and disruption of stacked GO domains, consistent with the PXRD studies.

**Figure 3:**
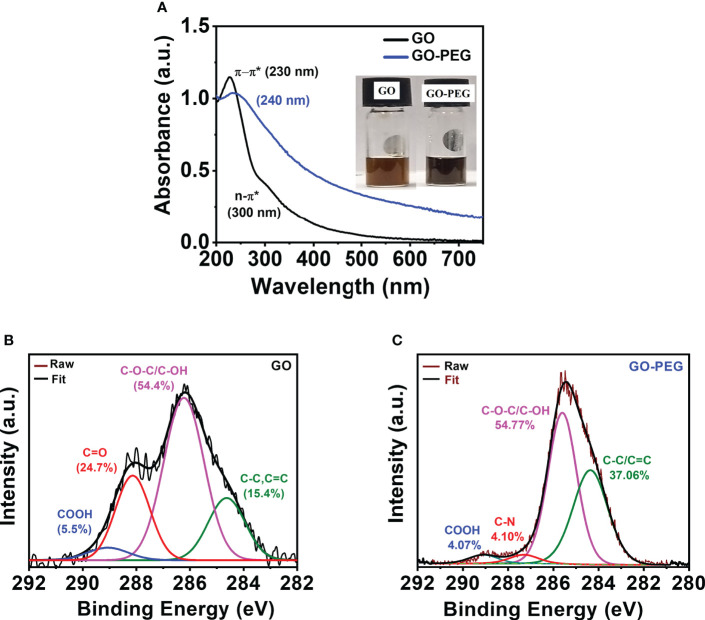
PEGylation of GO. **(A, B)** UV–visible spectra (inset shows digital images of the nanoparticle aqueous dispersions at a concentration of 0.5 mg/ml). XPS high-resolution deconvoluted spectra of C1s of **(B)** GO and **(C)** GO-PEG.

The UV–vis spectra of the aqueous dispersion of the synthesized nanomaterials are shown in **Figure 3A**. GO revealed two distinct absorption peaks at 230 nm and 300 nm, which originated from π–π^*^ transition of the conjugated C=C bond and the n-π^*^ transition of the >C=O bond, respectively (38). GO-PEG showed a red shift in the π–π^*^ transition from 230 nm to 240 nm with an undetectable shoulder at 300 nm, confirming that PEGylation of GO led to greater retention of conjugation in its basal plane. Additionally, optical images of the aqueous dispersions **(** **Figure 3A** **, inset)** clearly showed an alteration in color from red-brown in GO to dark-black in GO-PEG, which further supports the formation of a more conjugated structure in the latter.

XPS analysis was performed to determine chemical functionality and confirm successful functionalization of GO nanoparticles with PEG-amine. Deconvolution of the C1s XPS spectrum **(** **Figure 3B** **)** in GO revealed the existence of four main types of carbon bonds (binding energy) as C–C/C=C (284.5 eV), C–O/C–O–C (286.3 eV), C=O (288.1 eV) and COOH (289.0 eV) with relative percentages of 15.4%, 54.4%, 24.7%, and 5.5%, respectively. Similarly, the C1s spectra of GO-PEG were deconvoluted into four peaks **(** **Figure 3C** **)** as C–C/C=C (284.4 eV), C–O/C–O–C (285.7 eV), C–N (288.3 eV) and COOH (289.1 eV). Successful attachment of PEG to GO was confirmed by a simultaneous increase in the percentage of C–C/C=C to 37.06% and vanishing of >C=O XPS peak, and appearance of a new peak which is attributed to the C–N (of bound PEG-amine) with a relative percentage of 4.10%. This clearly demonstrated the base-mediated nucleophilic addition reaction of PEG-NH_2_ on the carbonyl groups in GO, consistent with the FTIR results.

**Figure 4:**
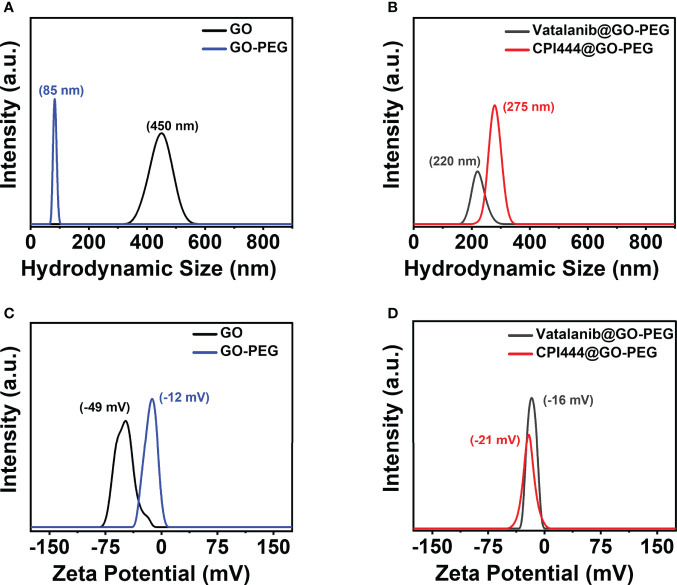
Dynamic Light Scattering measurements. **(A, B)** Hydrodynamic size, and **(C, D)** Zeta potential of GO, GO-PEG, vatalanib@GO-PEG and CPI444@GO-PEG, respectively.

The particle size and surface charge (zeta potential, ζ) of nanoparticles are critical parameters that dictate the interaction with the payload and may guide their cellular uptake. As can be seen in **Figures 4A, C**, the average hydrodynamic sizes (ζ) of GO and GP were determined as 450 nm (−49 mV) and 80 nm (−12 mV), respectively. PEG binding to GO substantially reduced the hydrodynamic size and reduction in negative charge (ζ) due to the positively charged amino groups of PEG, which counteracted the negatively charged carboxylate group (COO^−^) in GO. This may enable GO-PEG as an attractive nanocargo for intracellular delivery. The CPI444@GO-PEG and vatalanib@GO-PEG showed a hydrodynamic sizes of 275 nm and 220 nm, respectively **(** **Figure 4B** **)**. Drug loading on GO-PEG led to an increment in hydrodynamic size, clearly supporting the loading of hydrophobic drug molecules on GO-PEG nanoflakes. Further, a change in ζ value of GO-PEG in CPI444@GO-PEG and Vatalanib@GO-PEG showed (**Figure 4D** **)** a −21 mV and −16 mV, respectively, is noticed. Both zeta potential (ζ) and average hydrodynamic values of drug-nanoconjugates confirmed the successful loading of drug molecules on the GO-PEG nanocarrier.

### 3.2 Drug loading efficiency

**Figure 5:**
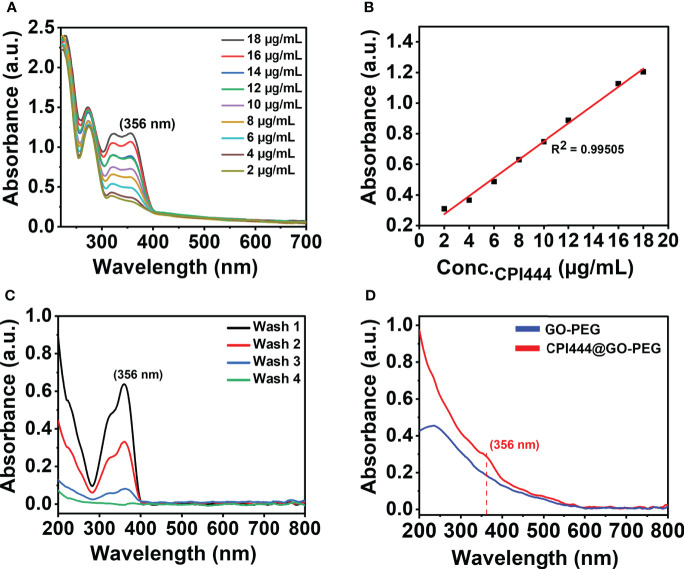
Quantitative and qualitative analysis for the formation of CPI@GO-PEG using UV–vis spectroscopy: **(A)** UV–vis spectra of CPI444 solution recorded at different concentrations in (water: THF) solvent mixture, **(B)** Linear equation obtained from the standard curve at a wavelength maximum of 356 nm, **(C)** UV–vis spectra recorded for unbound CPI444, **(D)** UV–vis spectra for GO-PEG and purified CPI444@GO-PEG.

The amount of drug loading on GO-PEG as in CPI444@GO-PEG and vatalanib@GO-PEG was determined spectrophotometrically. A standard curve was plotted by recording absorbance at a respective wavelength maximum using variable drug (CPI444, *λ*_max_ = 356 nm and vatalanib, *λ*_max_ = 335 nm) concentrations, and a linear good fit **(** **Figures 5A, B**, **Figures S1A, B** **)** was obtained. The unbound drug was removed by dialysis on the carrier and purification was monitored by UV–vis spectroscopy. As can be seen from **Figure 5C**, a subsequent decrease in absorbance at 356 nm in the dialysate is noticed to support leaching out of the loosely bound CPI444 on GO-PEG. The fourth washing showed the absence of any released CPI444, confirming the successful formation of a stable CPI444@GO-PEG conjugate. The CPI444 loading capacity and encapsulation efficacy were calculated as 55 wt% and 50 wt%, respectively. **Figure 5D** shows UV–vis spectra for GO and CPI444@GO-PEG, qualitatively confirming the loading of CPI44 onto the GO-PEG nanocarrier as it revealed the shoulder at 356 nm associated with bound CPI444 on GO-PEG. Likewise, the amount and existence of vatalanib on the GO-PEG nanocarrier was also determined. As can be seen in **Figure S1**, for vatalanib, the dialyzate revealed almost no release of the unbound drug, supporting its excellent adsorption on the GO-PEG nanocarrier. Structurally, vatalanib has a highly rigid aromatic framework compared to CPI444, which ensures relatively strong van der Waals interactions of the former with the carrier.

### 3.3 Improved cellular uptake of CPI444 and vatalanib *via* PEGylated graphene oxide nanocarrier resulting in enhanced cell death

The highly evasive nature of glioblastoma (GBM) is mainly due to the heterogeneity of the cellular and molecular players (3). This demands exploration of combinatorial nanotherapeutic approaches, i.e., utility of two or more drug molecules on a carrier to enhance high cellular uptake of drug and to induce pronounced effects in their specific roles to target different cellular mechanisms.

**Figure 6:**
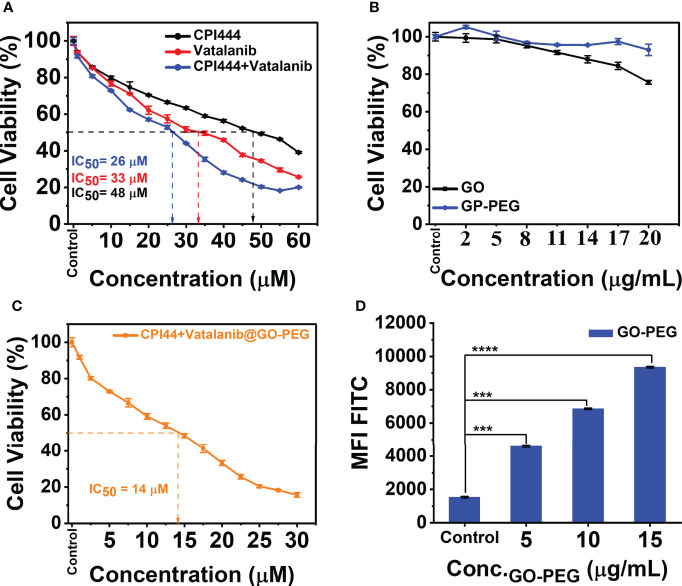
Cell viability studies and cellular uptake of PEGylated graphene oxide. **(A)** CPI444 and vatalanib cell cytotoxicity were calculated in GBM U87 cells. Cells were treated with drugs individually along with different concentrations for 48 h. After 48 h, IC_50_ values were calculated. **(B)** Cytotoxicity analyses of GO and GO-PEG were performed in GBM U87 cells. The PEGylated graphene oxide carrier did not show significant cytotoxicity in the GBM cell line up to 20 µg/ml. **(C)** In the GBM U87 cell line IC_50_ value of CPI444 + vatalanib@GO-PEG is ~14 µM. **(D)** To confirm the cellular internalization of PEGylated GO nanoparticles in the GBM cells, we conducted a FACS study after 24 h of incubation with different concentrations of PEGylated graphene oxide nanoparticles; MFI (Mean Fluorescence Intensity) in arbitrary units (AU); ****p <0.0001 and ***p <0.001.

CPI444 is an adenosine A_2A_ receptor antagonist emerging as a potential antitumor drug (20, 39, 40). Vatalanib is another potential drug that targets VEGFR tyrosine kinase and other class III kinases (41) and plays a significant role in targeting angiogenesis in GBMs (4). CPI444 and vatalanib have different molecular targets, enabling them to be suitable for GBM treatment. A nanocarrier, GO-PEG, was used to deliver both CPI444 and vatalanib drug molecules for better outcomes. Initially, a cell viability study was performed to assess the cytotoxic effects of CPI444, vatalanib, CPI444 + vatalanib, GO-PEG, and CPI444 + vatalanib@GO-PEG at different concentrations than individual drug molecules **(** **Figures 6A–C** **)**. The inhibitory concentration (IC_50_) values of CPI444, vatalanib, and in combination were determined as 48, 33, and 26 µM, respectively **(** **Figure 6A** **)**. The combination of CPI444 and vatalanib exhibited a drastic effect on cell survival at a relatively lower concentration than individual drug molecules. Further, the biocompatibility of the designed GO-PEG over GO was evaluated. It clearly revealed PEGylation substantially improved the cell viability up to 20 µg/ml, supporting it as a safe carrier **(** **Figure 6B** **)**. Finally, an IC_50_ value for the drug loaded GO-PEG (CPI444 + vatalani@GO-PEG) was determined as 14 µM **(** **Figure 6C** **)**. Drug molecules loaded with GO-PEG induced an elevated cytotoxic effect compared to the individual drug molecules or their combination. This assay suggests that the GO-PEG nanocarrier improves therapeutic efficacy without raising dosage. Additionally, the cellular uptake of GO-PEG nanoparticles in GBM U87 cells was calculated through flow cytometry **(** **Figure 6D**, **Figure S2** **)**. To perform this experiment, GO-PEG was tagged with FITC and incubated with the cells for 24 h in different concentrations (5 µg/ml, 10 µg/ml, and 15 µg/ml). Here, a concentration-dependent cellular uptake of GO-PEG-FITC was observed. Previously, GO-PEG has been shown to exhibit a minimal effect on cell viability in the range of ~50 µg/ml (42). This novel formulation of nanocarrier (GO-PEG) with CPI444 and vatalanib facilitated increased cellular uptake, thus allowing the effective reduction of inhibitory concentrations affecting cell viability. This will reduce significant side effects due to overdosage.

### 3.4 CPI444 and vatalanib loaded GO-PEG affect the proliferation and stemness properties of GBM U87 cells

**Figure 7:**
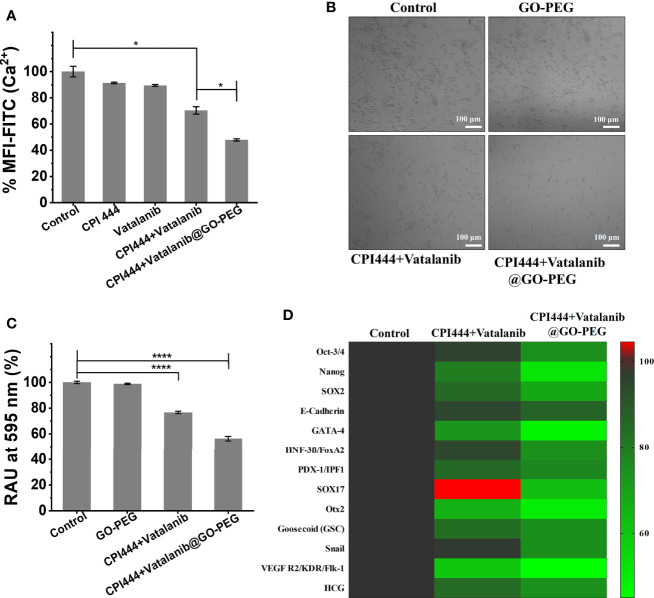
CPI444 and vatalanib-loaded GO-PEG affect the proliferation and stemness properties of GBM U87 cells. **(A)** Cellular calcium levels were estimated by FACS analysis using Fluo-4AM dye under different drug treatment conditions. **(B)** Colony forming assay of GBM U87 treated with untreated control, GO-PEG, CPI444 + vatalanib and CPI444 and vatalanib conjugated with GO-PEG. **(C)** Quantitation of colony forming assay, %RAU, relative absorbance units in percentage. **(D)** Heatmap depicting the expression levels of human pluripotent stem cell protein array for pluripotency and differentiation markers upon treatment with CPI444 and vatalanib loaded on GO-PEG with appropriate controls. MFI (Mean Fluorescence Intensity) in arbitrary units (AU); ****p <0.0001 and *p <0.05.

Glioblastoma multiforme (GBM) is a type of high-grade primary brain cancer that comprises a subpopulation called glioblastoma stem cells (GSC), which are responsible for tumor initiation, development, and recurrence following treatment (3). Calcium (Ca^2+^) is a secondary messenger that plays a vital role in cell proliferation, cell death, differentiation, migration, metabolism, neural plasticity, and gene transcription, among other processes (43, 44). Calcium signaling is reported to have a significant impact on neuronal cell development and function (45). Glioblastoma is of neuronal origin, and therefore, calcium pathways are active in controlling and promoting various cellular functions (46). Therefore, intracellular calcium (Ca^2+^) levels were assessed in GBM U87 cells upon treatment with CPI444 and vatalanib-loaded PEGylated graphene oxide nanoparticles. Here, a significant reduction in Ca^2+^ levels was observed in CPI444 + vatalanib and CPI444 + vatalanib@GO-PEG treated cells compared to untreated or cells treated with either of the drugs individually **(** **Figure 7A**, **Figure S3** **)**. A 2D colony forming assay was performed to identify the role of reduced intracellular calcium (Ca^2+^) in the inhibition of GBM stem cell properties. A combination of nanoconjugated CPI444 and vatalanib reduced the colony formation in GBM U87 cells **(** **Figures 7B, C** **)**. This indicates that reduced cellular calcium levels affect the proliferation ability of glioblastoma stem cells (GSCs). A human pluripotent stem cell array analysis was performed under similar treatment conditions to further investigate the molecular targets that are affected. Here, markers for pluripotency such as Oct3/4, Nanog, and Sox2 exhibit significant downregulation upon treatment with CPI444 and vatalinib loaded on GO-PEG **(** **Figure 7D** **)**. Additionally, markers associated with the epithelial to mesenchymal transition (EMT), such as Snail and Goosecoid (Gsc), as well as other markers for differentiation to other cell lineages exhibited downregulation **(** **Figure 7D** **)**. This suggests that the GSCs are not undergoing differentiation into assume other cell fates.

These observations suggest that combinatorial treatment of CPI444 with vatalanib with the aid of GO-PEG is effective in reducing the cellular calcium pool of GBM U87 cells. This is a very promising observation as Ca^2+^ levels play a critical role not only in GSC proliferation and in glial cell function that helps in the survival and fate determination process (44, 47).

### 3.5 GBM U87 cells migration and invasive potential is compromised by combinatorial treatment of CPI444 and vatalanib loaded on GO-PEG

**Figure 8:**
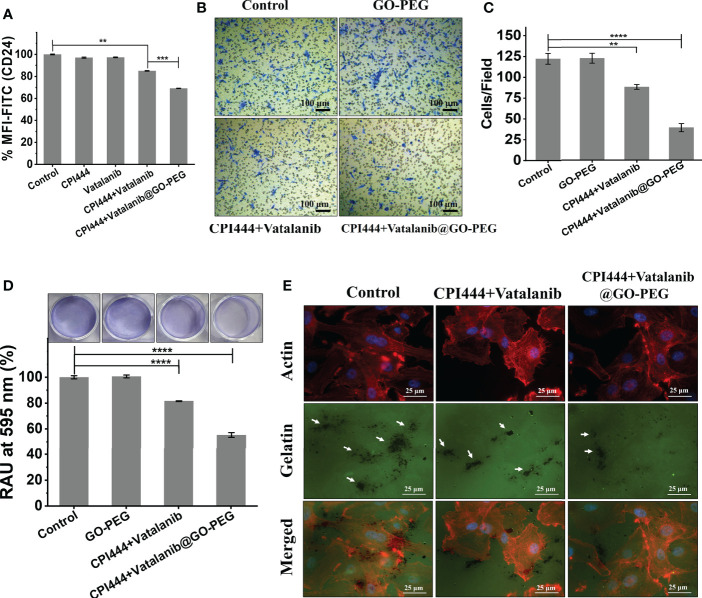
CPI444 and vatalanib@GO-PEG hindered the cellular migration and invasive behavior of GBM U87 cells. **(A)** CD24 expression levels in GBM U87, quantitated by FACS analysis after treatment with CPI444 and vatalanib-loaded GO-PEG and other combinations. **(B, C)** Boyden chamber analysis (Transwell migration assay) was performed for cell migration properties. The combination of nanoconjugate CPI444 and vatalanib-treated cells shows smaller numbers of migrated cells compared to control. (**D)** The adhesive property of GBM cells was significantly reduced after the treatment with the GO-PEG conjugated combination of CPI444 and vatalanib. RAU%, Relative absorbance units in percentage. (**E)** Invadopodia causing dark gelatin degradation patches (indicated by white arrows) were detected by the gelatin degradation assay under different treatment conditions. ****p <0.0001, ***p <0.001, and **p <0.01.

Glioblastoma multiforme is an aggressive tumor that has an aggressive migratory property and invades the microenvironment and adjacent brain tissue. CD24, a motility and invasion marker, was overexpressed in gliomas, particularly in GBM (48–50). Therefore, to assess the effect of CPI444 and vatalanib loaded GO-PEG on CD24 expression in GBM U87 by FACS analysis is therefore necessary. A significant reduction in the CD24 expression was observed **(** **Figure 8A**, **Figure S4** **)**. Further, the migration abilities of GBM U87 cells under similar treatment conditions were estimated by the Boyden chamber assay. Here, a drastic reduction in the cell numbers that have migrated to the Boyden chamber was observed **(** **Figures 8B, C** **)**. Cell motility is linked to adhesive properties; an important reason for the decreased migration is the attenuation of adhesion. Therefore, we assessed the adhesive property of GBM U87 cells upon treatment with the CPI444 + vatalanib@GO-PEG along with the comparable controls and observed reduced cellular adhesion compared to the control conditions **(** **Figure 8D** **)**. Finally, gelatin degradation assay was performed after treatment with CPI444 + vatalanib@GO-PEG, CPI444 + vatalanib, and compared with the untreated cells as a control. A visible reduction in the dark patches formed by invadopodia during cell migration in nanoparticles mediated drug-treated cells was evident **(** **Figure 8E**). This clearly demonstrates a profound impairment in the cellular migration of GBM U87 cells.

In the previous section, a drastic reduction in the cellular Ca^2+^ levels was observed upon treatment with CPI444 + vatalanib@GO-PEG. There are ever-growing studies suggesting the role of calcium-mediated signaling in cell migration during development and in cancer metastasis (51, 52). Previous reports have also shown that CD24 plays a critical role in the invasive properties of cancer/tumor cells (48, 50). Therefore, we hypothesize that the observed reduction in expression of CD24, along with the non-invasive phenotype of GBM U87, upon treatment with CPI444 and vatalanib *via* GO-PEG, could be due to reduced Ca^2+^ levels.

### 3.6 Treatment with CPI444, vatalanib, and CPI444 +vatalanib@GO-PEG caused reduced secretion of angiogenic factors and apoptosis of GBM U87 cells

**Figure 9:**
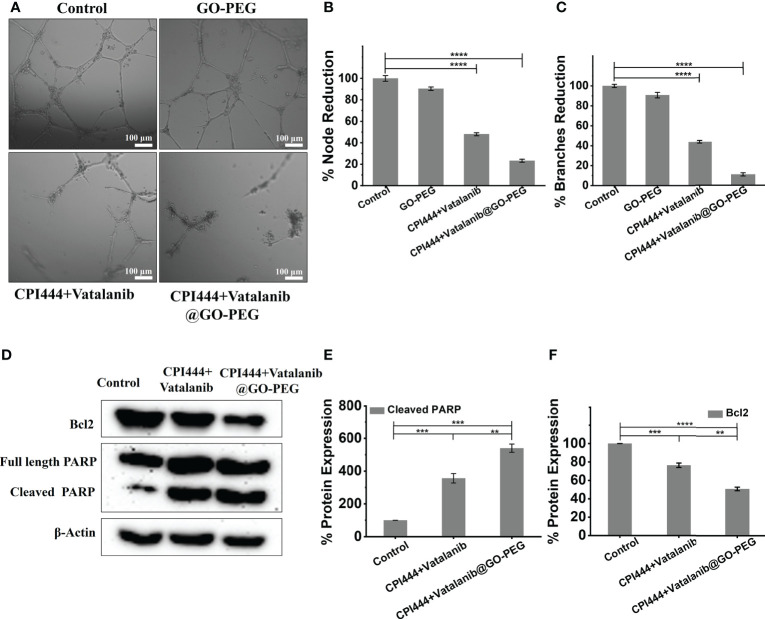
Treatment of CPI444 and vatalanib-loaded GO-PEG inhibits the angiogenic potential and causes apoptosis of GBM U87. **(A–C)** A tube formation assay was performed for angiogenesis analysis in GBM cells. GBM U87 cells showed a smaller number of nodes and branches compared to control cells. **(D–F)** Western blot analysis of Bcl2, intact PARP, cleaved PARP. β-actin was used as an internal control. The Western blot shown is a representative of three independent experiments. ****p <0.0001, ***p <0.001, and **p <0.01.

The glioblastoma tumor microenvironment exhibits characteristic vascularization mediated by various growth factors and conditions. GBM U87 cells secrete particular growth factors that cause neo-angiogenesis in pre-existing capillaries (49). We analyzed the effect of CPI444 and vatalanib loaded GO-PEG on the angiogenic potential of GBM U87. The essential developmental molecules for tube formation are found in the tissue culture media (TCM) of GBM U87 cells. The TCM of treated and untreated GBM U87 cells was collected and provided to the primary endothelial cells for tube formation. The TCM of control cells revealed a rich and progressive vasculature and showed a larger number of branches and nodes compared to the treated cells **(** **Figures 9A–C** **)**. Previous reports have shown that vatalanib exhibits anti-angiogenic properties in multiple cancers, including tumors (53, 54). Purinergic signaling has also been shown to help in tumor neovascularization by inducing the secretion of VEGF into the tumor microenvironment (55, 56). Therefore, a combinatorial treatment with GO-PEG mediated high cellularity proves to be very effective in the inhibition of angiogenic signals from GBM U87 cells. The observed morphology also explains why VEGF2R was downregulated upon treatment with CPI444 + vatalanib@GO-PEG **(** **Figure 7D** **)**.

So far, in this study, multiple assays have indicated that the loss of stemness properties leads to reduced proliferation capacity upon CPI444 + vatalanib@GO-PEG treatment. At the same time, GBM U87 cells were also not differentiated into any specialized cell types **(** **Figures 7D**, **9A–C** **).** This indicates that the cells might be exhibiting arrested growth or undergoing programmed cell death. To understand this, the expression of apoptotic markers such as the proteolytic cleavage product of poly (ADP-Ribose) polymerase (PARP) and B-cell lymphoma 2 (Bcl2) was scored **(** **Figures 9D–F** **)**. Here, upregulated cleaved PARP and downregulation of Bcl2 clearly demonstrate that combinatorial treatment with improved cellular uptake results in apoptosis of GBM U87 cells.

## 4 Conclusion

In this study, a nanocarrier GO-PEG was synthesized and characterized. PEGylation of graphene oxide resulted in a significant reduction in hydrodynamic size, and zeta potential, which facilitated a high cellular uptake of GO-PEG. Additionally, PEGylation of graphene oxide led to reduced cytotoxicity. This nanocarrier was used to deliver potential therapeutic drug molecules, CPI444 and vatalanib, to target GBM U87 cells derived from glioblastoma multiforme. These drugs were mainly selected to target divergent cellular mechanisms as GBM exhibits molecular and cellular heterogeneity to its advantage. This study clearly demonstrates that the stem cell properties of GBM U87 were severely affected by custom-designed nanoformulation. Additionally, the treatment with CPI444 and vatalanib *via* GO-PEG led to a drastic reduction in the metastatic ability of GBM U87 cells. The current work has proven the importance of the novel formulation under study in the effective reduction of the angiogenic potential of GBM. Ultimately, this nanosized CPI444 and vatalanib caused elevated apoptosis, which will lead to a reduced tumor size. This nanoparticle-based combinatorial therapy approach enhances the efficacy of the existing therapeutic drugs by targeting the key molecular players at very low concentrations. This property will provide a huge advantage in tackling drug resistance exhibited by tumors and avoiding off-target effects due to overdosage.

## Data availability statement

The original contributions presented in the study are included in the article/**Supplementary Material**. Further inquiries can be directed to the corresponding authors.

## Author contributions

BL conceived the concept and designed the chemical structures. PR designed the biological assays. SP performed the chemical synthesis, and characterization of nanoparticles. VM performed the biological assays. BL and PR interpreted the data. VM and SP made equal contributions. All authors listed have made a substantial, direct, and intellectual contribution to the work and approved it for publication.

## Funding

BL and PR are thankful for the financial support received from the Shiv Nadar Foundation. PR is also supported by the Early Career Fellowship of the Wellcome Trust-DBT India Alliance (IA/E/16/1/503057).

## Acknowledgments

VM and SP acknowledge the fellowship received from Shiv Nadar University. The authors thank Dr. Seema Sehrawat for the initial discussion. The authors would like to thank Dr. Sri Krishna Jayadev Magani (SNU, Delhi-NCR) for his kind gift of PARP and Bcl2 antibodies.

## Conflict of interest

The authors declare that the research was conducted in the absence of any commercial or financial relationships that could be construed as a potential conflict of interest.

## Publisher’s note

All claims expressed in this article are solely those of the authors and do not necessarily represent those of their affiliated organizations, or those of the publisher, the editors and the reviewers. Any product that may be evaluated in this article, or claim that may be made by its manufacturer, is not guaranteed or endorsed by the publisher.
