# Supplementary material for: Combinatorial delivery of CPI444 and vatalanib loaded on PEGylated graphene oxide as an effective nanoformulation to target glioblastoma multiforme: In vitro evaluation
Source: Front Oncol. 2022 Aug 16;12:953098. doi: 10.3389/fonc.2022.953098 (PMC9426685; doi:10.3389/fonc.2022.953098)
Supplement: Supplementary file 1 [file DataSheet_1.pdf]

Combinatorial Delivery of CPI444 and Vatalanib Loaded on PEGylated Graphene Oxide as an Effective Nanoformulation to Target Glioblastoma Multiforme: In vitro Evaluation

Vishnu S. Mishra,[1†] Sachin Patil,[2†] Puli Chandramouli Reddy,[*,1] and Bimlesh Lochab[*,2]

[1]Department of Life Sciences, School of Natural Sciences, Shiv Nadar University, Delhi-NCR, 201314, India.

[2]Materials Chemistry Laboratory, Department of Chemistry, School of Natural Sciences, Shiv Nadar University, Delhi-NCR, 201314, India.

*Corresponding authors: Puli Chandramouli Reddy (pc.reddy@snu.edu.in); Bimlesh Lochab (bimlesh.lochab@snu.edu.in)

Figure S1. Quantitative and qualitative analysis for formation of Vatalanib@GO-PEG using UV-vis. spectroscopy: (a) UV-vis. spectra of Vatalanib solution recorded at different concentrations in (water: THF) solvent mixture, **(b)** Linear equation obtained from standard curve at wavelength maximum at 335 nm, (c) UV-vis. spectra recorded for unbound Vatalanib, **(d)** UV-vis. spectra for GO-PEG and purified Vatalanib@GO-PEG.

Figure S2. Cellular uptake of PEGylated Graphene Oxide Nanoparticles was confirmed and validated by FACS study.

Figure S3. Intracellular calcium ions determined by FACS analysis.

Figure S4. CD24 expression analysis in GBM cells by FACS.

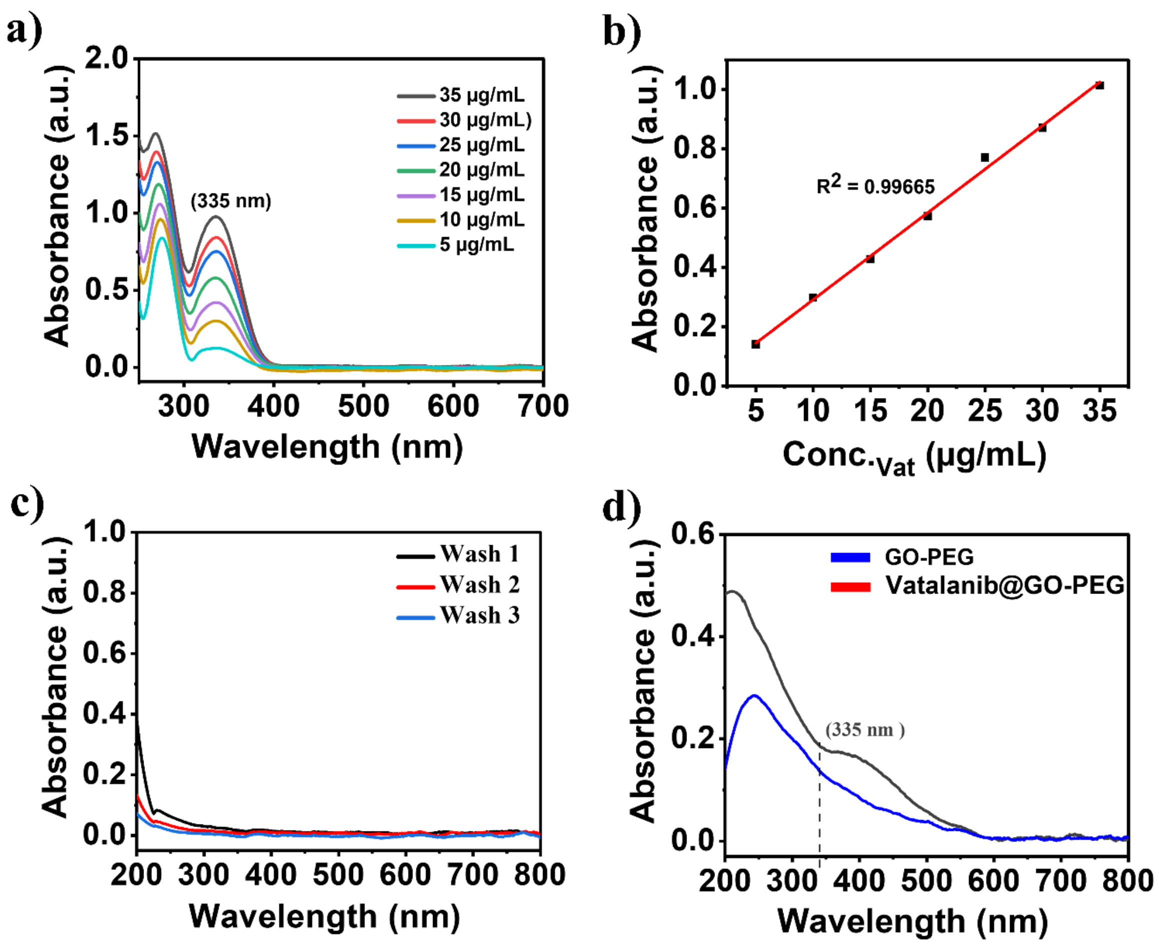

Figure S1. Quantitative and qualitative analysis for formation of Vatalanib@GO-PEG using UV-vis. spectroscopy: (a) UV-vis. spectra of Vatalanib solution recorded at different concentrations in (water: THF) solvent mixture, **(b)** Linear equation obtained from standard curve at wavelength maximum at 335 nm, (c) UV-vis. spectra recorded for unbound Vatalanib, **(d)** UV-vis. spectra for GO-PEG and purified Vatalanib@GO-PEG.

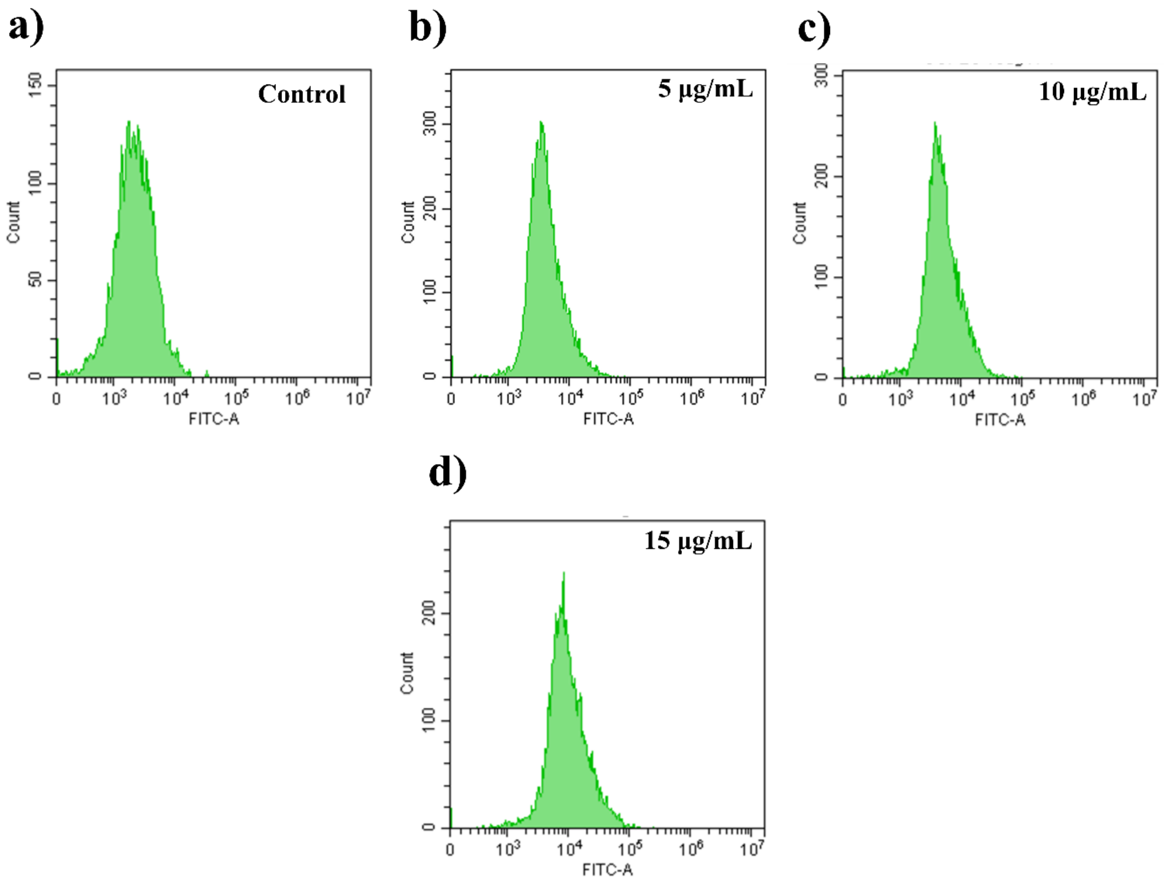

Figure S2. Cellular uptake of PEGylated Graphene Oxide Nanoparticles was confirmed and validated by FACS study.

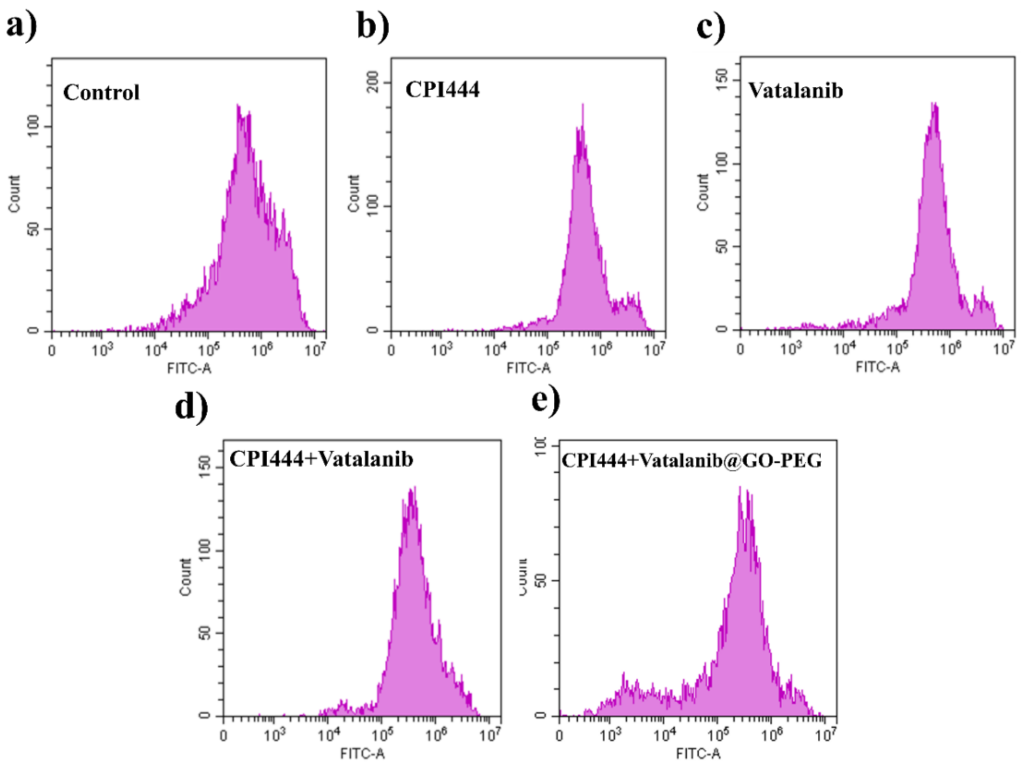

Figure S3. Intracellular calcium ions determined by FACS analysis.

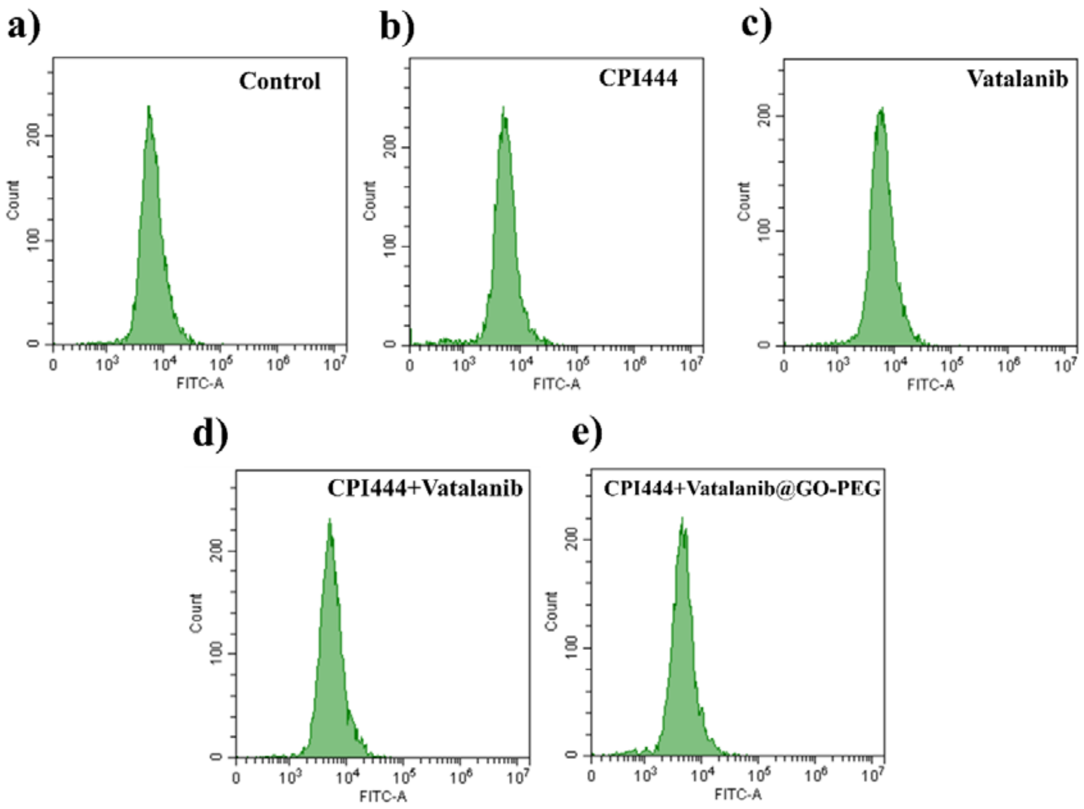

Figure S4. CD24 expression analysis in GBM cells by FACS.
