# Supplementary figures and images for: Combinatorial delivery of CPI444 and vatalanib loaded on PEGylated graphene oxide as an effective nanoformulation to target glioblastoma multiforme: In vitro evaluation
Source: Front Oncol. 2022 Aug 16;12:953098. doi: 10.3389/fonc.2022.953098 (PMC9426685; doi:10.3389/fonc.2022.953098)

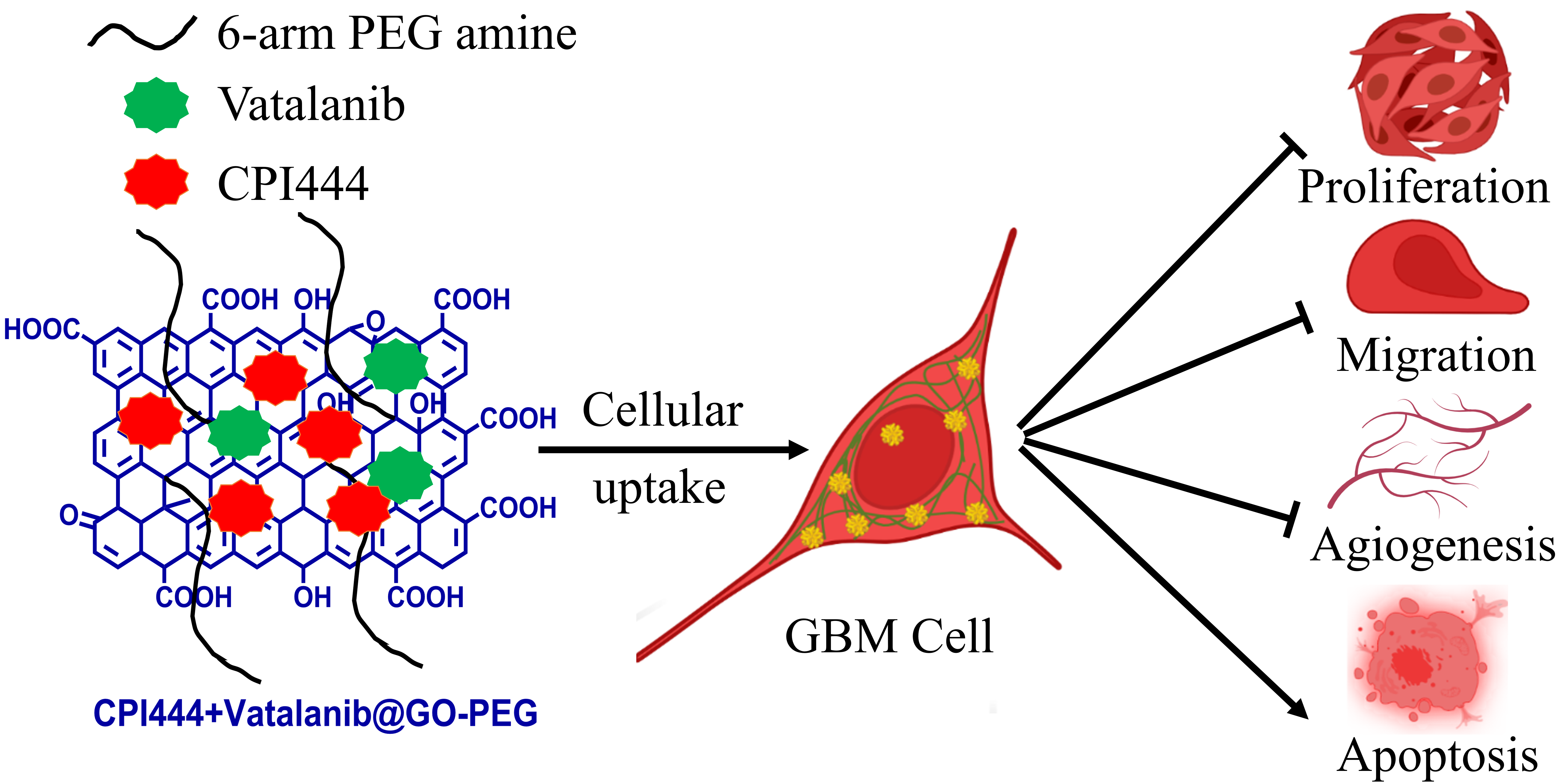

Supplement: Supplementary file 2 [file Image_1.tif]
